# Precision Treatment of Pulmonary Sarcoma Metastasis Using Personalized Ultrafractionated Stereotactic Adaptive Radiotherapy: A Case Report

**DOI:** 10.7759/cureus.82084

**Published:** 2025-04-11

**Authors:** Anastasia Stergioula, Theodoros Kormas, Stefania Kokkali, Anastasios Kyriazoglou, Evaggelos Pantelis

**Affiliations:** 1 Department of Radiotherapy, Iatropolis Clinic, Athens, GRC; 2 Department of Orthopedic Surgery, Agios Savvas Anticancer Hospital, Athens, GRC; 3 Department of Oncology, Ippokrateio General Hospital of Athens, Athens, GRC; 4 Second Propaedeutic Department of Medicine, Attikon University Hospital, National and Kapodistrian University of Athens, Athens, GRC; 5 School of Medicine, National and Kapodistrian University of Athens, Athens, GRC

**Keywords:** cyberknife, pulsar, sbrt, soft tissue sarcoma, srt

## Abstract

The lungs represent the most common sites of distant metastases in soft tissue sarcoma (STS) patients. The relative radioresistance of STS renders them ideal targets for stereotactic radiotherapy (SRT). In this study, the treatment of a complex STS lung metastases case involving complete main bronchus occlusion and lung collapse using an image-guided, personalized ultrafractionated stereotactic adaptive radiotherapy (PULSAR) approach is reported. A biologically effective dose (BED) of 102 Gy_10_ was delivered in two stages separated by 21 days using the CyberKnife^TM^ platform (Accuray Inc., Sunnyvale, CA, USA) and the Synchrony Lung Optimized (Synchrony-LOT^TM^) motion management system (Accuray Inc.). Each treatment stage was based on real-time imaging data, allowing for the adaption of the treatment plan to the tumor's size and shape. Prior to the second stage, significant tumor regression was observed, leading to lung re-expansion and restoration of pulmonary function. This expansion enabled the visualization and treatment of a second peripheral lesion, which received a BED of 106 Gy_10_ in a single session. The applied treatment protocol resulted in excellent local control and minimal toxicity. The combination of the PULSAR approach and real-time imaging techniques hold significant promise for treating complex cases and marks a shift toward more adaptive and personalized radiation oncology.

## Introduction

In stereotactic radiotherapy (SRT), a highly conformal dose distribution is registered to a well-defined target within the body. This technique delivers an increased dose to the target over a short treatment course, resulting in an enhanced biologically effective dose (BED) and improved local disease control [1]. To minimize complications, small (if no) margins should be applied around the target, whereas SRT dose distributions should be characterized by steep spatial dose gradients to spare the surrounding healthy tissues from increased doses. SRT has been proven particularly effective for treating radioresistant tumors (e.g., melanoma, renal cell carcinoma) characterized by low α/β ratio values. Soft tissue sarcoma (STS) tumors are considered particularly radioresistant, requiring increased doses to achieve local control [2]. The treatment of primary STS combines surgery and radiotherapy (RT). Approximately half of STS patients eventually develop distant metastases, with the lungs being one of the most common sites [3,4]. SRT for STS lung metastases has shown excellent local control rates (85% to 99% at two years follow-up) and minimal toxicity profiles [5-7]. The dose prescription strategy is typically based on the size and location of the lesions with regard to critical structures, with smaller and peripheral targets treated with 30 Gy in a single fraction, while larger targets or targets located in the ultra-central zone treated with 60 Gy in 8 fractions in an effort to minimize toxicity [5].

This study outlines the treatment protocol and clinical outcome of an STS lung metastases case, treated with a two-staged ultrafractionated SRT approach utilizing the CyberKnife^TM^ robotic radiosurgery platform (Accuray Inc., Sunnyvale, CA, USA) to alleviate lung collapse caused by complete obstruction of the main bronchus by the lesion.

## Case presentation

**Figure 1:**
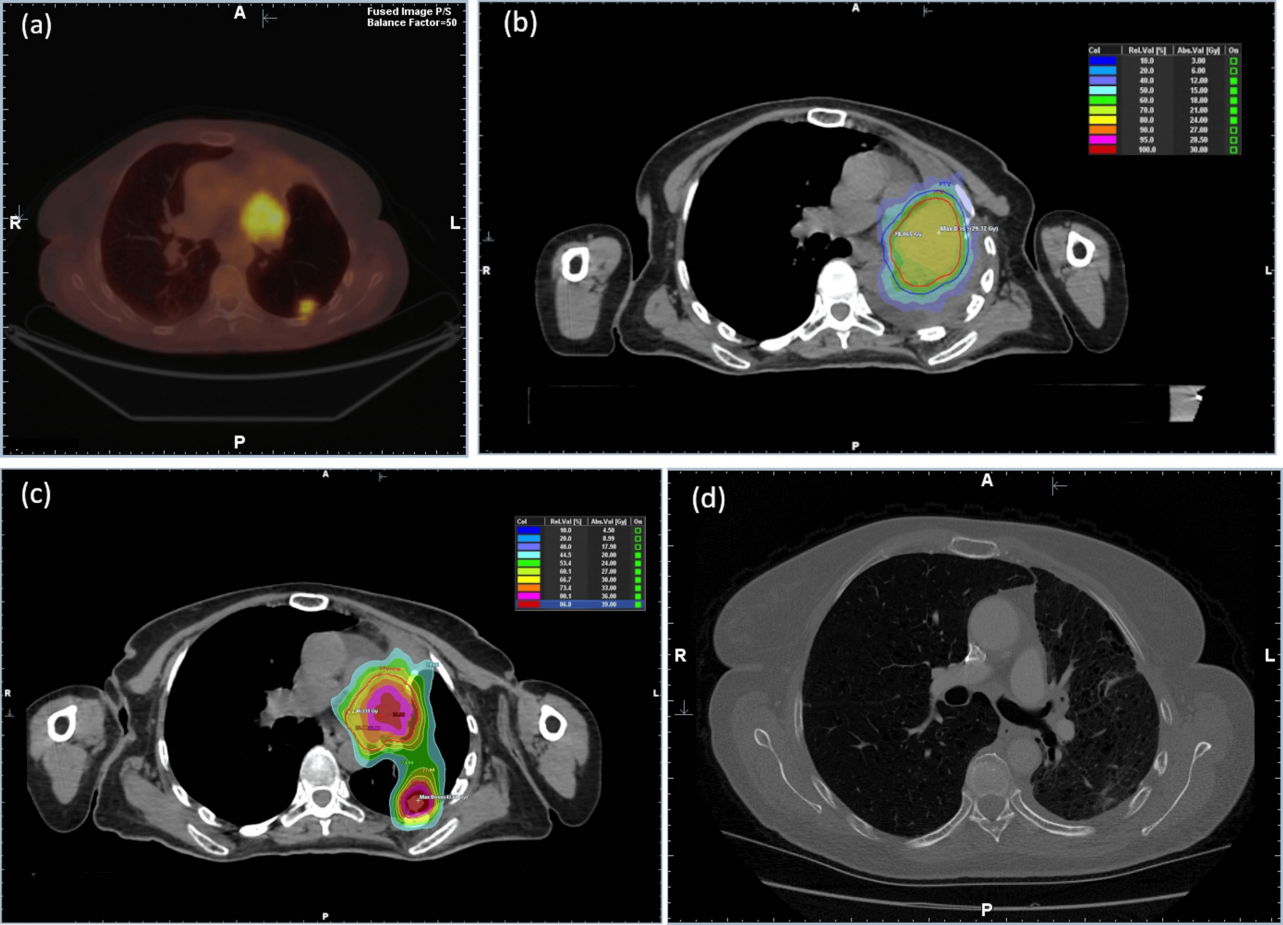
Imaging and treatment overview. (a) Axial PET-CT image of the studied case showing two soft tissue sarcoma metastases in the hilar and superior lower lobe of the left lung. (b) Axial simulation CT image acquired 15 days post PET-CT scan showing left lung complete occlusion. A two-stage SRT technique was followed for the hilar lesion. The dose distribution of the first stage overlayed on the simulation CT is shown. (c) Axial simulation CT image performed 21 days after the first stage. The summed dose distribution from the two-stage treatment of the hilar lesion and the single fraction treatment of the peripheral lesion is shown. (d) Axial follow-up CT image with contrast enhancement of the patient nine months post-treatment showing excellent response. PET-CT: positron emission tomography-computed tomography; SRT: stereotactic radiotherapy

A patient with a primary malignant peripheral nerve sheath tumor (MPNST) of the left thigh, managed with surgery and pre-operative chemo-radiotherapy, presented with a single lung metastasis. The metastasis was treated with surgical resection followed by systemic therapy. On follow-up positron emission tomography-computed tomography (PET-CT) scan acquired 11 months post-surgery, local recurrence was detected along with a new peripheral lung metastasis (Figure 1a). The patient was considered to be in an oligometastatic state [8] and referred for SRT to treat the two lung lesions.

On the simulation CT scan, it was found that the lung metastasis in the hilar completely obstructed the left main bronchus, resulting in left lung collapse (Figure 1b). The patient was asymptomatic. The hilar lesion could be identified on the simulation CT, whereas the peripheral lesion could not be visualized. A personalized ultrafractionated stereotactic adaptive radiotherapy (PULSAR) [9] approach was used to treat the patient, delivering the therapeutic dose to the hilar lesion in two stages. The goal was to achieve tumor regression, reopen the bronchus, expand the left lung to spare healthy lung tissue, and restore the patient’s respiratory capacity. All treatments were performed using the CyberKnife platform and the Synchrony Lung Optimized Tracking (Synchrony-LOT^TM^) (Accuray Inc.) motion management system [10].

The hilar lesion could not be identified by the stereoscopic kilovoltage (kV) X-ray based target locating system (TLS) in both treatment stages and therefore the *0-view* option of the Synchrony-LOT system was used (i.e., the nearest three vertebrae were tracked and used to determine the location of the lesion during treatment delivery) [10]. Treatment planning was performed using free breathing and non-contrast-enhanced simulation CT images of the patient’s treated anatomy. A four-dimensional CT (4DCT) scan with contrast enhancement media was acquired prior to each stage to quantify target movements. The internal target volume (ITV) was delineated with the aid of maximum image projection (MIP) CT images, obtained from the patient’s 4DCT scan, along with the PET-CT imaging data registered to the simulation CT images through deformable registration methods available in the Precision^TM^ treatment planning system (TPS) (Accuray Inc.). The planning target volume (PTV) was defined by expanding the ITV isotropically by 3 mm. In the first stage, a marginal dose of 18 Gy was delivered in a single fraction to the hilar lesion, with the dose to the main bronchus restricted to less than 15 Gy (Figure 1b). A diagnostic CT scan was acquired 21 days post first-stage treatment, revealing a reduction of the hilar lesion and expansion of the left lung. Consequently, a new simulation CT and 4DCT scans were performed on the same day and used for the planning of the second stage of the treatment (Figure 1c). Target contour was adapted using the new planning CT and 4DCT imaging datasets. A dose of 27 Gy in three fractions was delivered on alternate days to the hilar lesion using the *0-View *Synchrony-LOT option, resulting in a total dose of 45 Gy. The expansion of the left lung allowed for the visualization of the second peripheral lung metastasis, which was treated with a single fraction of 28 Gy in a separate treatment session. The motion of the lesion could be tracked by the TLS, and therefore, the *2-view* option of the Synchrony-LOT system was used [10]. The TPS Monte Carlo dose calculation algorithm was used for dosimetric calculations in all treatment plans.

In Figure 1c, the dose summation of all SRT plans - calculated using mutual information-based deformable registration algorithms - is overlaid on the axial CT images acquired for the second stage. The total BED values were found to be equal to 102 Gy_10 _and 106 Gy_10_ for the hilar and peripheral lung lesion, respectively. Furthermore, using an α/β ratio of 5 Gy, as reported by Haas et al. [11], the BED values for the hilar and peripheral lung lesions were calculated to be equal to 159 Gy_5_ and 185 Gy_5_, respectively. Finally, using the described PULSAR approach, a maximum main bronchus BED value - defined at a volume of 35 mm^3^ - of less than 175 Gy_3_ was achieved, minimizing toxicity risk [12]. Maximum BED values of 34 Gy_2_ and 33 Gy_3_ were also calculated for the spinal cord and esophagus organs at risk, respectively.

The SRT regimen resulted in a marked tumor response as shown on follow-up imaging at the nine-months post-treatment CT scan (Figure 1d). No significant radiation-induced pneumonitis or severe adverse events were observed.

## Discussion

An STS lung metastases case presented with complete occlusion of the left main bronchus resulting in ipsilateral lung collapse is reported. The peripheral lesion in the left lung could not also be visualized. The metastasis obstructing the bronchus was treated using a PULSAR approach, which involved delivering an increased BED to the lesion in two stages, separated by a 21-day interval. This approach allowed for tumor regression, reopening of the main bronchus and restoration of pulmonary function. By adapting the treatment plan based on the tumor’s response, excellent local disease control was achieved with minimal toxicity.

Spreading the fractions of an SRT course over multiple days, with intervals of at least 10 days, has shown promising results in preclinical models [9], and is currently being evaluated for treating brain metastases [13], non-small cell lung cancer (NSCLC), metastatic colorectal cancer and palliative head and neck treatments [14]. This approach, coined as PULSAR, essentially optimizes cancer treatment by delivering highly targeted doses while adapting to tumor changes over time and potentially enhancing both tumor radiosensitivity and immune response. Each treatment session is personalized based on real-time imaging, tumor dynamics, and patient response, ensuring optimal therapeutic efficacy. In the presented case, although not all treatment fractions were equally spaced, the applied PULSAR approach resulted in an excellent outcome. Furthermore, the expansion of the lung also allowed the visualization and treatment of the second peripheral lesion.

Minimization of target margins is critical to all SRT treatments [1]. For lesions in the lung in particular, advanced treatment delivery systems should be used to accommodate for the motion of the target due to respiration [1]. These systems include (a) dynamic shifting of the treatment beam to follow target motion, (b) dynamic shifting and reshaping of the multileaf collimator (MLC) aperture to follow target motion, (c) gaiting techniques that turn the beam on/off based when target is inside/outside predefined thresholds, and (d) abdomen compression techniques to minimize target motion. In the presented case, the use of dynamic beam shifting with target motion was not possible in the first stage of the treatment due to the left lung collapse. In the second stage, where the left lung was re-expanded, real-time tracking of both lesions was evaluated on a lung-optimized tracking simulation session. The hilar lesion could not be identified on the stereoscopic kV X-ray images. Therefore, the *0-view* Synchrony-LOT motion management system was used to accommodate patient movements in both treatment stages. A PTV margin of 3 mm was added to the ITV to account for residual treatment delivery uncertainties. In contrast, lung expansion allowed tracking of the peripheral lesion, which was treated using the *2-view* Synchrony-LOT system. This system enabled the dynamic shifting of each treatment beam with target motion, eliminating the need for an ITV and minimizing the margins around the target. A PTV margin of 3 mm around the second target was used.

## Conclusions

The use of PULSAR for the treatment of a complex STS lung metastases case involving complete obstruction of the left lung main bronchus causing lung collapse is reported. Delivering the SRT dose in two stages, separated by 21 days, with adaptation for tumor regression, led to the restoration of pulmonary function and achieved excellent local control with minimal toxicity. It also allowed for the visualization and successful treatment of the second peripheral lung metastasis. The combination of PULSAR and real-time imaging techniques holds significant promise for the treatment of complex cases and represents a shift toward more adaptive and personalized radiation oncology.
